# Pathogenomic Characterization of Multidrug-Resistant *Escherichia coli* Strains Carrying Wide Efflux-Associated and Virulence Genes from the Dairy Farm Environment in Xinjiang, China

**DOI:** 10.3390/antibiotics14050511

**Published:** 2025-05-15

**Authors:** Muhammad Shoaib, Sehrish Gul, Sana Majeed, Zhuolin He, Baocheng Hao, Minjia Tang, Xunjing Zhang, Zhongyong Wu, Shengyi Wang, Wanxia Pu

**Affiliations:** 1 Key Laboratory of New Animal Drug Project Gansu Province, Key Laboratory of Veterinary Pharmaceutical Development, Ministry of Agriculture and Rural Affairs, Lanzhou Institute of Husbandry and Pharmaceutical Sciences of Chinese Academy of Agricultural Sciences, Lanzhou 730050, Chinazhuolinhe0902@163.com (Z.H.); haobaocheng@caas.cn (B.H.); tangminjia@outlook.com (M.T.); xuejingzhang123@126.com (X.Z.); wu-zhongyong@outlook.com (Z.W.); wangshengyi@caas.cn (S.W.); 2 Jiangsu Co-Innovation Center for Prevention and Control of Important Animal Infectious Diseases and Zoonoses, College of Veterinary Medicine, Yangzhou University, Yangzhou 225009, China; 3 Institute of Microbiology, University of Agriculture, Faisalabad 38000, Pakistan; sehrishgulsg@gmail.com; 4 Laboratory of Aquatic Animal Medicine, College of Veterinary Medicine, Chungbuk National University, Cheongju 28644, Republic of Korea; majeedsana@chungbuk.ac.kr

**Keywords:** *Escherichia coli*, antibiotic efflux genes, virulence genes, serotyping, prophages, genomic islands, bacterial secretions systems, biofilm-associated genes

## Abstract

**Background/Objectives**: Livestock species, particularly dairy animals, can serve as important reservoirs of *E. coli*, carrying antibiotic resistance and virulence genes under constant selective pressure and their spread in the environment. In this study, we performed the pathogenomic analysis of seven multidrug resistant (MDR) *E. coli* strains carrying efflux-associated and virulence genes from the dairy farm environment in Xinjiang Province, China. **Methods**: First, we processed the samples using standard microbiological techniques followed by species identification with MALDI-TOF MS. Then, we performed whole genome sequencing (WGS) on the Illumina NovaSeq PE150 platform and conducted pathogenomic analysis using multiple bioinformatics tools. **Results**: WGS analysis revealed that the *E. coli* strains harbored diverse antibiotic efflux-associated genes, including conferring resistance to fluoroquinolones, aminoglycosides, aminocoumarins, macrolides, peptides, phosphonic acid, nitroimidazole, tetracyclines, disinfectants/antiseptics, and multidrug resistance. The phylogenetic analysis classified seven *E. coli* strains into B1 (*n* = 4), C (*n* = 2), and F (*n* = 1) phylogroups. PathogenFinder predicted all *E. coli* strains as potential human pathogens belonging to distinct serotypes and carrying broad virulence genes (ranging from 12 to 27), including the Shiga toxin-producing gene (*stx1*, *n* = 1). However, we found that a few of the virulence genes were associated with prophages and genomic islands in the *E. coli* strains. Moreover, all *E. coli* strains carried a diverse bacterial secretion systems and biofilm-associated genes. **Conclusions**: The present study highlights the need for large-scale genomic surveillance of antibiotic-resistant bacteria in dairy farm environments to identify AMR reservoir spillover and pathogenic risks to humans and design targeted interventions to further stop their spread under a One Health framework.

## 1. Introduction

*Escherichia coli* (*E. coli*) is the most common inhabitant of the intestinal tracts of humans and animals. Most *E. coli* strains are harmless, but some can cause diseases in humans and animals [1]. Livestock species can serve as key reservoirs of antibiotic-resistant and human pathogenic *E. coli*, including the Shiga toxin-producing *E. coli* (STEC) [2]. STEC is a prevalent foodborne human pathogen worldwide, leading to over one million infections and more than 100 fatalities annually [3]. Most *E. coli* strains are classified into the phylogroups A, B1, B2, and D, differing in phenotypic and genotypic traits, ecological niches, adaptability, and disease-causing potential [4]. Additionally, there are minor phylogroups, such as C, E, F, and the cryptic clade I [5]. The B1 group, notable for its ability to persist in the environment, differs from the others in ecological niches and antibiotic resistance [6]. However, there is limited information about the geographic distribution, host preferences, phenotypic and genotypic characteristics, or disease-causing potential of minor phylogroups [5].

Antimicrobial resistance is a dynamic, multifaceted issue influenced by many diverse factors [7]. Unjustified usage of antimicrobial agents in agricultural and animal settings has contributed greatly to the emergence of multidrug resistance (MDR) traits within animal-derived bacteria [8]. Resistance determinants evolve in environmental niches due to the horizontal transfer of mobile genetic elements (MGEs) across bacterial species, even without direct antimicrobial pressure [9]. *E. coli* is an important indicator of antimicrobial resistance in various animals because of its role as a common gut commensal and occasional pathogen and its high capacity for acquiring antibiotic resistance genes (ARGs). *E. coli* can serve as a reservoir, transferring resistance genes to other bacteria [10]. *E. coli* can acquire antimicrobial resistance because of selective pressure in various environments and hosts [11,12]. *E. coli* strains are resistant to multiple antibiotics in animals and humans, including tetracyclines, aminoglycosides, phenicols, streptomycin, erythromycin, carbapenems, cephalosporins, sulfonamides, and β-lactams [12]. Antibiotic-resistant *E. coli* can transfer from livestock to humans and the environment through farm waste disposal and manure applications [13]. The ARGs found in human pathogenic *E. coli* may be similar to the environmental *E. coli* of dairy farms, including manure from dairy cows, which may indicate their spread from the environment to clinical pathogens [14].

Shiga toxin virulence markers determine the *E. coli* strains as STEC. There are two different genes responsible for Shiga toxin production, called *stx1* and *stx2*, which are usually located on lambdoid prophages and reflect the mobile nature of the *stx* genes. STEC can acquire virulence-related genes by horizontal gene transfer from pathogenic bacteria [15]. Other accessory virulence factors involved in pathogenicity are *eae* (encoding intimin for enterocyte effacement), *tir* (translocated intimin receptor), *espAB* (encoding for needle complex type III secretion system), and *hylA* or *ehxA* (encoding hemolysin) [15]. Another virulence factor that plays a role in antibiotic resistance is biofilm formation. Biofilm is the accumulation of bacterial cells on the surface enveloped by a protective extrinsic polymeric matrix [16,17]. Biofilms are significant factors contributing to the long-term survival of *E. coli* in food processing plants, since they are pervasive and resist disinfection [18].

Furthermore, mobile genetic elements (MGEs), including plasmids, integrons, and others, can be found in different bacteria and play an important role in spreading the virulence and antibiotic resistance genes within and between bacterial pathogens [19,20]. There is a need to characterize pathogenic traits such as virulence genes, serotypes, secretion systems, and biofilm-associated genes of antibiotic-resistant *E. coli* in dairy farm environments, which was previously a neglected horizon. **Dairy farm environments**, despite their known role as reservoirs for antibiotic-resistant bacteria, have been **understudied** compared to clinical or human and pig farm-associated settings in China. The present study was designed to conduct the in-depth genomic analysis of *E. coli* strains from the dairy farm environment in Xinjiang Province, China, with the primary aim of identifying ARGs, virulence genes, and phylogenetic analysis through whole genome sequencing (WGS) to assess the potential reservoir of ARGs, their pathogenic potential, and relationship with other strains, respectively.

## 2. Results

### 2.1. Cluster of Orthologous Genes (COG) Functional Classification of MDR E. coli

The COG functional classification revealed that *E. coli* strain 18XJ85 carried the highest number of genes (*n* = 4736), followed by 19XJ31 (*n* = 4647), 18XJ24 (*n* = 4572), 18XJ28 (*n* = 4485), 17XJ28 (*n* = 4460), 17XJ30 (*n* = 4455), and 17XJ31 (*n* = 3951) (Table 1). All of the *E. coli* strains carried *n* = 2 RNA processing and modification genes except 18XJ85 (*n* = 3). Additionally, we found that a large number of genes were involved in the transport and metabolism of various substances, such as nucleic acids, amino acids, carbohydrates, lipids, inorganics, coenzymes, and secondary metabolites. Furthermore, our analysis revealed that numerous functional genes contribute to multiple cellular processes, such as energy production and conversion, cell cycle, transcription, translation, posttranslational modifications, cell wall synthesis, cell membrane synthesis, and various defense mechanisms. Additionally, the present study strains had a large number of unknown function genes (functional class S) and mobilome genes (functional class X) (Table 1). The *E. coli* strains 18XJ85 and 17XJ31 carried the highest number of unknown function genes (*n* = 272) and mobilome genes (*n* = 218), respectively. In contrast, Table 1 presents the detailed distribution of functional genes belonging to each class for every *E. coli* strain.

### 2.2. Phylogenetic Analysis and Distribution of Antibiotic Efflux-Associated Genes

**Figure 1:**
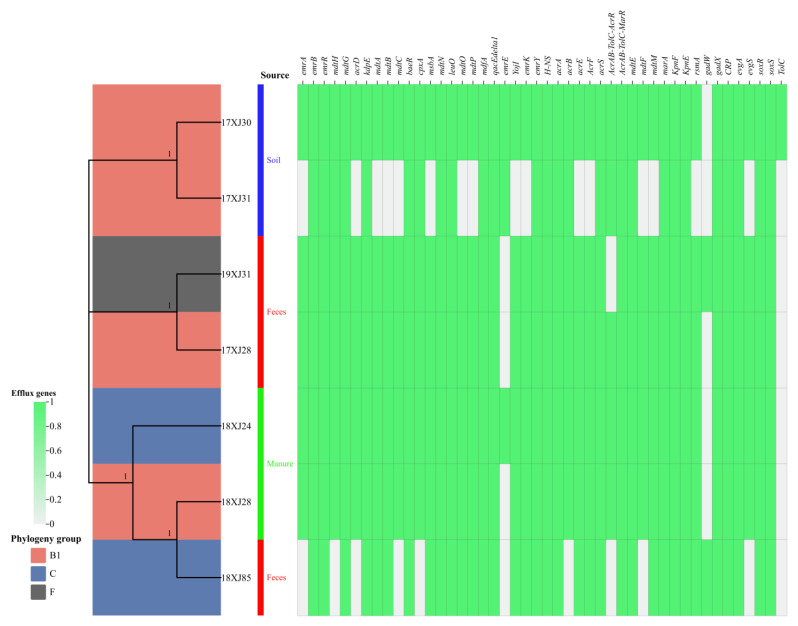
Phylogenetic analysis, isolation source, and heatmap of distribution of antibiotic efflux-associated genes of seven MDR *E. coli* strains. Levels 0 (grey color) and 1 (green color) indicate the absence and presence of the gene, respectively.

This study recovered seven *E. coli* strains carrying MDR efflux-associated genes from the dairy farm environment in Xinjiang Province, China [9]. In silico Clermont typing classified the seven *E. coli* strains isolated from soil (*n* = 2), feces (*n* = 3), and manure (*n* = 2) into three phylogenetic groups: B1 (*n* = 4), C (*n* = 2), and F (*n* = 1) (Figure 1). The 7 strains acquired a total of 46 different efflux-associated genes responsible for resistance to many antibiotic classes. The *E. coli* strain 17XJ30 was carrying the highest number of efflux-associated genes (*n* = 45), followed by 18XJ28 (*n* = 44), 17XJ28, 18XJ28, and 19XJ31 (*n* = 43 each), 18XJ85 (*n* = 35), and 17XJ31 (*n* = 28). This study identified several antibiotic efflux-associated genes, such as *emrR*, *emrB*, *mdtG*, *kdpE*, *baeR*, *mdtN*, *leuO*, *mdfA*, *qacE-delta1*, *emrY*, *H-NS*, *acrA*, *acrS*, *acrAB-TolC-marR*, *marA*, *kpnE*, *kpnF*, *gadX*, *CRP*, *evgA*, *soxS*, and *soxR* genes, belonging to different antibiotic classes. The distribution of other identified genes was as follows: *mdtH*, *mdtA*, *mdtB*, *cpxA*, *msbA*, *mdtO*, *mdtP*, *YojI*, *emrK*, *acrB*, *acrE*, *acrF*, *mdtM*, and *rsmA* in 6/7 strains and *emrA*, *acrD*, *mdtC*, *AcrAB-TolC-AcrR*, *mdtF*, and *evgS* in 5/7 strains, while *emrE*, *gadW*, and *TolC* were identified in 3/7, 2/7, and 1/7 strains, respectively (Figure 1).

### 2.3. Serotyping, CH-Typing, and Virulence Determinants Acquired by MDR E. coli

**Figure 2:**
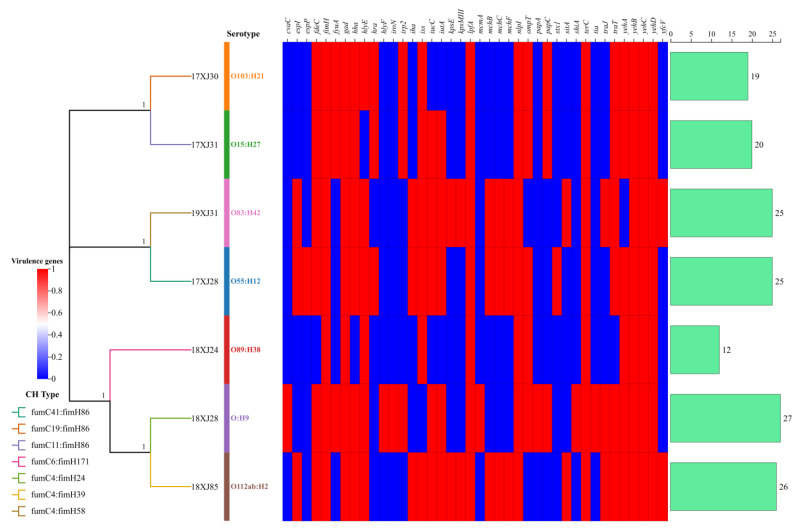
Serotyping, CH typing, and heatmap of distribution of virulence genes of seven MDR *E. coli* strains. Levels 0 (blue color) and 1 (red color) indicate the absence and presence of the virulence gene, respectively. The bar graph shows the number of virulence genes acquired by each strain.

The serotyping and CH typing analysis revealed that the seven *E. coli* strains belonged to distinct serotypes and CH types carrying diverse virulence genes. The identified serotypes were O55:H12, O103:H21, O15:H27, O89:H38, O novel:H9, O112ab:H42, and O83:H42 (Figure 2). Generally, the seven *E. coli* strains carried in the range of 12–27 virulence genes. The *E. coli* strain 18XJ28 was carrying the highest number of virulence genes (*n* = 27), followed by 18XJ85 (*n* = 26), 17XJ28 and 19XJ31 (*n* = 25 each), 17XJ31 (*n* = 20), 17XJ30 (*n* = 19), and 18XJ24 (*n* = 12). Notably, *E. coli* strain 17XJ28 carries the Shiga toxin-producing gene (*stx1*) belonging to the O55:H12 serotype and fumC41: fimH86 CH-type (Figure 2). PathogenFinder predicted that all of the *E. coli* strains are potential human pathogens.

### 2.4. Phylogenetic Relationship of E. coli Strains with Verified E. coli Pathotypes

**Figure 3:**
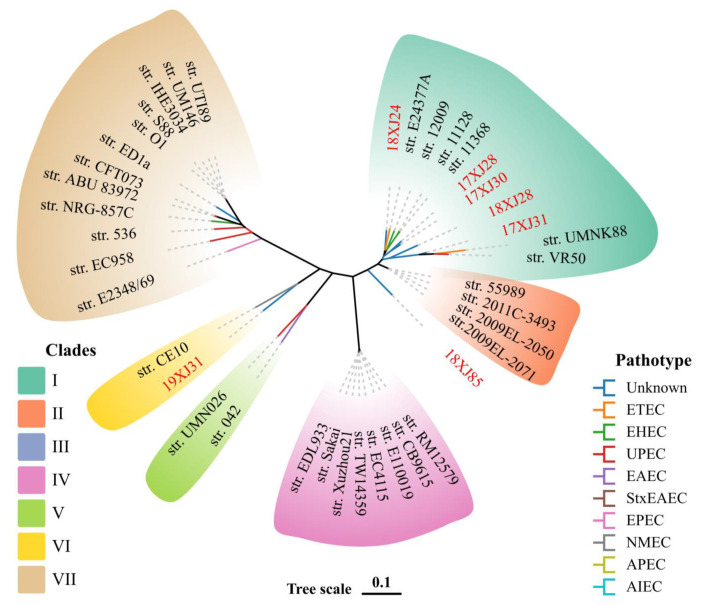
Phylogenetic relationship of seven MDR *E. coli* strains with verified *E. coli* pathotypes from the Virulence Factor Database based on core-genome SNPs. The seven clades are marked with different colors, while the tree branch colors show the pathotype distinction.

Our study performed a phylogenetic analysis of seven *E. coli* strains with verified *E. coli* pathotypes to reveal the evolutionary relationship and genetic similarities. The phylogenetic analysis classified all *E. coli* strains into seven clades (clades I–VII). The clade I carried 11 *E. coli* strains (five from this study and six other *E. coli* pathotypes). Clade II was carrying *n* = 4, clade III *n* = 1, clade IV *n* = 8, clade V *n* = 2, clade VI *n* = 2, and clade VII *n* = 12 *E. coli* strains (Figure 3). The *E. coli* strain 18XJ85 showed little genetic similarity with other pathotypes included in this study, and we classified it into a separate clade III. Moreover, the *E. coli* strain 19XJ31 showed increased genetic similarity with the neonatal meningitis-causing *E. coli* (NMEC) strain CE10 and classified it under clade VI (Figure 3).

### 2.5. Prophage and Genomic Island-Associated Virulence Genes (VGs)

The prophage analysis of seven *E. coli* strains revealed that six VGs (*stx1*, *colE5*, *hha*, *iha*, *iss*, and *ompT*) were associated with the prophage genome of *E. coli* strain 17XJ28, three VGs (*hra*, *iss*, and *papC*) with 17XJ30, three (*f17G*, *f17C*, and *nlpI*) with 17XJ31, one (*iss*) with 18XJ24 and 18XJ85, one (*mcmA*) with 18XJ28, and three (*iss*, *nlpI*, and *terC*) with 19XJ31 prophage genome segments. The *iss* gene was found in 5/7 and *nlpI* in 2/7 prophage genomes, while other VGs were identified in a single *E. coli* strain (Table 2). We identified *fimH* and *ompT* as genomic island-associated genes in the *E. coli* strain 18XJ24. The *afaA*, *afaB*, *afaC*, *afaD*, *iha*, and *kpsE* genes were associated with the genomic island of the 19XJ31. However, none of the VG was associated with the genomic island of 17XJ28, 17XJ30, 17XJ31, 18XJ28, and 18XJ85 strains (Table 2).

### 2.6. Bacterial Secretions Systems Related Genes Acquired by MDR E. coli Strains

**Figure 4:**
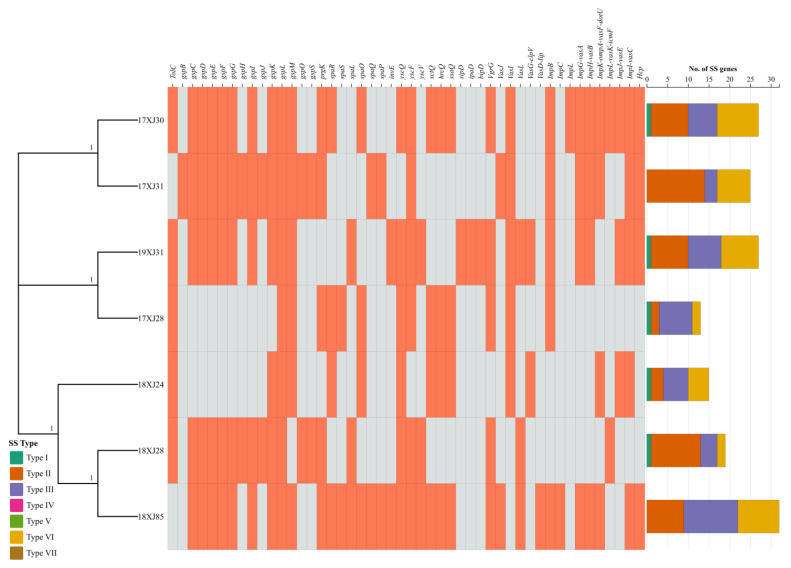
Heatmap of the distribution of different types of secretion system genes acquired by seven MDR *E. coli* strains. The grey and orange color indicates the absence and presence of the secretion system gene, respectively. The stacked bar graph shows the number of secretion system genes belonging to a specific type carried by each strain. Current study strains did not identify types IV, V, and VI SS genes; therefore, the bar graph does not show color related to these types.

All of the seven *E. coli* strains carried at least one of the types 2, 3, and 6 secretion system (SS) genes, while five strains carried type 1 SS genes, except 17XJ31 and 18XJ85. Five *E. coli* strains carried only one type 1 SS gene. However, type 2, 3, and 6 SS genes were acquired by all *E. coli* strains, varying from 2–21, 2–9, and 4–13, respectively. The *E. coli* strain 18XJ85 acquired the highest number (*n* = 32) of SS genes, followed by 17XJ30 and 19XJ31 (*n* = 27), 17XJ31 (*n* = 25), 18XJ28 (*n* = 19), 18XJ24 (*n* = 15), and 17XJ28 (*n* = 13) (Figure 4). We identified the *gspL* gene (belonging to type 2 SS) in all *E. coli* strains. In contrast, Figure 4 presents the distribution of other SS genes belonging to different types.

### 2.7. Biofilm-Associated Genes Acquired by MDR E. coli Strains

All *E. coli* strains isolated from the dairy environment carried a large set of biofilm genes, including genes activated by environmental signals. Four *E. coli* strains (17XJ30, 17XJ31, 18XJ28, and 18XJ85) carried a total of *n* = 48 biofilm genes, while three *E. coli* strains (17XJ28, 18XJ24, and 19XJ31) carried a total of *n* = 46 biofilm genes. The four *E. coli* strains (17XJ30, 17XJ31, 18XJ28, and 18XJ85) carried two additional genes, *dksA* and *ag43* (Table 3). However, none of the *E. coli* strains carried sRNAs involved in biofilm formation.

## 3. Discussion

*E. coli* is a robust organism and survives in all environments, even outside of a host organism. However, a deeper understanding of the diversity and adaptability of these strains in various environments is needed. The present study characterized seven MDR *E. coli* strains through whole-genome sequencing. In this study, in silico Clermont typing revealed that most of the *E. coli* strains belonged to phylogroup B1, carrying wide resistance and virulence genes [21]. Another study reported that *E. coli* strains isolated from milk, farm workers, and environmental settings also carried resistance, virulence, and mobilome genes [21,22]. *E. coli* belonging to the B1 phylogroup has increased adaptability compared to other groups in different hosts [23,24,25]. Alternatively, it could be due to phylogroup B1’s better survival outside hosts due to its distinctive stress tolerance traits [23]. Rehman et al. [14] also reported the presence of *E. coli* in manure belonging to the B1 phylogroup. Consistent with our study, *E. coli* isolated from the animal feces belonging to the B1 and F phylogroups have also been reported [26,27].

Efflux pumps are crucial for *E. coli* to resist multiple drugs, and their presence or absence significantly impacts the resistance profile of each strain. The efflux-associated genes that mainly contribute to MDR are *mdtE*, *mdtF*, *mdtM*, *marA*, *kpnF*, *rsmA*, *gadW gadX*, *CRP*, *evgA*, *evgS*, *soxR*, *soxS*, *tolC*, and other antibiotics like fluoroquinolones (*emrA*, *emrB*, *emrR*, and *mdtH*), phosphonic acid (*mdtG* and *acrD*), aminoglycosides (*acrD*, *kdpE*, *mdtA*, *mdtB*, and *mdtC*), aminoglycosides and aminocoumarins (*baeR* and *cpxA*), nitroimidazoles (*msbA*), nucleoside and disinfecting agents (*mdtN*, *leuO*, *mdtO*, *mdtP*, and *qac-E-delta*), tetracycline, nucleoside and disinfecting agents (*mdfA*), macrolides (*emrE*), glycopeptides (*YojI*), tetracycline (*emrK*, *emrY*, and *mdfA*) [28]. The *AcrAB-TolC* is an operon that naturally makes *E. coli* resistant to carbonyl-cyanide m-chlorophenylhydrazone (CCCP) and nalidixic acid and is associated with an MDR efflux pump [29]. Although 18XJ28, 18XJ24, and 17XJ28 strains lack the *gadX* and *TolC* genes. However, they have *AcrAB-TolC-AcrR* and *AcrAB-TolC-MarR* tripartite efflux systems, which enable them to exhibit MDR phenotype. These settings are known for high microbial diversity and antibiotic pressures, which can drive the selection of resistant strains [29].

The identification of the *gadX* gene in only two fecal strains in our study suggests the potential role in acid resistance mechanisms among these *E. coli* strains and is critical for E. coli survival in low-pH environments, such as the stomach. The absence of the *gadX* gene in the soil and other environmental *E. coli* strains suggests a decreased Na+ concentration, reduced acid resistance in soil and environment, and downregulated expression of *gadE*, *gadA*, *gadB*, and *gadC* efflux pumps [30]. This information is crucial for understanding the adaptive strategies of *E. coli* in surviving extreme gastric acidity before colonizing the intestine [30]. The absence of *TolC* in all strains except one soil *E. coli* strain suggests either compensatory mechanisms or niche-specific adaptations in these strains [31]. Strains may utilize TolC-independent pumps, such as EmrAB, MdtABC, which were found in most *E. coli* strains. Strains 18XJ85 and 19XJ31, collected from feces, have fewer efflux-associated resistance genes compared to others, which may reflect their specific ecological niche or selective pressures in the environment from which they were isolated [32,33]. Continuous monitoring and characterization of bacterial strains are crucial to identify and exploit such vulnerabilities for better treatment strategies.

The CH and serotype analysis provided insights into these strains’ virulence potential and colonization abilities. The fimbrial gene (*fimH*) associated with the type of adherence system was present in all strains in our study, contributing to their virulence and ability to adhere to host tissues, which is crucial for establishing infections and biofilm formation [1]. The present study identified that Shiga toxin-producing *E. coli* (STEC) belong to the flagellar H12. *E. coli* belonging to the O55:H12 serotype have also been identified in environmental, human, and animal samples earlier [34,35,36]. The single STEC strain identified in our study may represent a high-risk outlier or indicate horizontal gene transfer events within the farm environment. In the present study, the 17XJ30 strain isolated from soil belongs to the O103:H21 serotype. O103 is included in one of the “big six” serotypes identified by the FDA and is most frequently associated with foodborne sickness and diarrhea in different countries [37,38]. O103:H21 serotype has also been identified as the verotoxigenic *E. coli* (VTEC) pathotype from bovine carcass [39]. Brusa et al. [40] identified *E. coli* belonging to the O103:H21 serotype from cattle slaughterhouses. *E. coli* strain 17XJ31, isolated from the soil of a dairy environment, belongs to the O15:H27 serotype. Egervärn and Flink have reported that the O15:H27 serotype causes food borne illness [41]. Galarce et al. [42] also reported the O15:H27 serotype from the livestock–food–human interface. Van Overbeek et al. reported that *E. coli* belonging to the O89:H38 serotype was isolated from manure [43]. *E. coli* O112ab:H2 serotype was detected in the ileal microbiota of a wild boar suffering from diarrhea and carried *hlyE*, *lpfA*, and *gad* genes [44]. The *E. coli* O83:H42 serotype is known to be responsible for urinary tract infections [45]. Moreover, this study noted a high prevalence of *tia*, *espI*, *esp*, *yeh*, and *hylE* genes in these environmental strains. The *yeh* fimbrial loci affect gene expression and virulence in enterohemorrhagic *E. coli* O157 [46]. The higher number of virulence genes acquired by these strains suggests a strong potential for pathogenicity, highlighting the need for monitoring and control strategies for these pathogens to prevent outbreaks.

Prophages and genomic islands frequently play a role in host survival strategies and enhance the genetic diversity of the host genome [47]. Additionally, prophages act as vehicles for the horizontal transfer of antimicrobial resistance and virulence genes [48]. Virulence factor genes in prophages enhance phage infectivity and are often involved in superinfection exclusion, contributing to host virulence. These genes likely benefit the prophage more than the bacteria, reducing selective pressure for the prophage to become defective [49]. The many prophages found in the sequenced genomes support that these mobile genetic elements (MGEs) significantly contribute to the evolution and genetic diversity of *E. coli* pathotypes [50]. The avian pathogenic *E. coli* (APEC) strains most frequently carry the increased serum survival (*iss*) and *ompT* virulence genes. The *iss* gene is crucial for resistance against serum complement, with *E. coli* producing *iss* proteins to achieve this complement resistance [51]. The *OmpT* gene detected in *E. coli* isolated from feces and manure in our study encodes the *OmpT* protein that serves as an antigen in STEC. This protease plays a recognized role in the virulence of extraintestinal pathogenic *E. coli* (ExPEC), APEC, and diarrheagenic *E. coli* (DEC) strains [52,53]. Fimbriae/adhesins related to the *papC* gene have also been identified in calves that have umbilical infection [54]. The presence of specific virulence genes (VGs), such as *stx1*, *colE5*, *hra*, *F17G*, *F17C*, *mcmA*, *iss*, *nlpI*, and *terC*, suggests that these strains have acquired virulence factors through prophage integration. Transmission of *stx* genes through prophage integration has also been reported earlier, which supports the findings of the present study [55]. Identifying a wide range of virulence genes and their specific distributions among the isolates provides valuable insights into the genetic diversity and adaptability of *E. coli* isolates in different environments.

In Gram-negative bacteria, extracellular enzyme secretions occur through either a one-step or a two-step process. In the two-step process, proteins first cross the cytoplasmic membrane into the periplasm [56]. The second step involves transporting these proteins across the outer membrane using specialized systems known as type II and type V secretion systems (T2SS and T5SS, respectively). In contrast, the one-step process employs type I (T1SS), type III (T3SS), type IV (T4SS), and type VI (T6SS) secretion systems, which transport proteins directly from the cytoplasm to the outside environment, bypassing the periplasm [57,58].We detected T2SS, T3SS, and T6SS in all isolates in our study. While T1SS was absent in one soil and one feces isolate, genes encoding T6SS were identified in mastitis-causing *E. coli* [59]. T6SS-related *imp* genes have been identified in *Klebsiella* spp. [60].

Under stress, *E. coli* forms biofilms by halting flagellar synthesis and producing curli fimbriae and extracellular polysaccharides controlled by the *csgBA* and *csgDEFG* operons. The master regulator *csgD* governs these operons and other growth-related genes, with its expression regulated by environmental factors and transcription factors [61]. In our study, all the isolates contain a high number of genes responsible for biofilm formation, taken from different dairy environments, which may indicate the possible threat of these strains. The present study has not performed in vitro, in vivo, or clinical trials to check the pathogenicity of characterized strains, which can be the future horizon for further research. However, the present study demonstrated that *E. coli* can be a potential reservoir of antibiotic resistance efflux, virulence, and biofilm genes in a dairy environment, which needs immediate attention to prevent their further spread and infection by these strains.

## 4. Materials and Methods

### 4.1. Origin and Background Information of E. coli Strains

**Figure 5:**
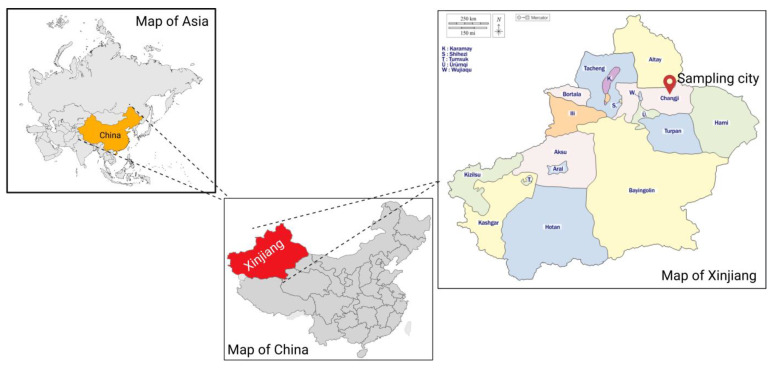
Illustration of regional (Asia), country (China), and province (Xinjiang) level maps to show sampling location. The location pin indicates the sampling city.

The seven *E. coli* strains included in this study were isolated from the dairy farm environment samples, including soil (17XJ30 and 17XJ31), manure (18XJ24 and 18XJ28), and cattle feces (17XJ28, 18XJ85, and 19XJ31) collected from Changji Hui Autonomous Prefecture in Xinjiang Province, China (map is illustrated in Figure 5). Briefly, a total of 209 samples, including feces, manure or slurry, water, milk, and soil, were collected from selected dairy farm, and 338 *E. coli* strains were retrieved, comprising 67.5% (141/209) of the collected samples. The antimicrobial susceptibility testing of retrieved *E. coli* strains revealed that 84.0% (284/338) were resistant to at least one antimicrobial, and 26.0% (54/338) were susceptible. The selection of these seven *E. coli* strains was completed based on co-carrying a gene array: *bla*_OXA-1_-*catB3*-*arr3* genes, whose detailed information about sampling protocols, processing, and selection criteria has been described in earlier published studies [1,9]. Briefly, the samples were enriched in Luria Bertani broth and then streaked on MacConkey agar. The isolated red- or pink-colored colonies were further streaked on Eosin Methylene Blue (EMB) agar for selective differentiation. The metallic green sheen colonies were picked for species identification. Matrix-assisted laser desorption/ionization time of flight mass spectrometry (MALDI-TOF MS) confirmed *E. coli* as done earlier [62]. Bacterial liquid cultures were stored at −80 °C in 20% glycerol for further processing and long-term storage.

### 4.2. DNA Extraction, Library Construction, and Whole Genome Sequencing (WGS)

The genomic DNA of seven *E. coli* strains was extracted using the sodium dodecyl sulfate (SDS) method for longer DNA fragments. The purity and integrity of extracted DNA were determined by agarose gel electrophoresis and quantification using a Qubit 4.0 fluorometer (Thermo Fisher Scientific, Waltham, MA, USA). The purified and integrated DNA fragments were randomly interrupted by a Covaris ultrasonic beaker (Woburn, MA, USA) with 350 bp inserts length. Following the manufacturer’s guidelines, the library was prepared using the NEBNext^®^ UltraTM DNA library kit for Illumina NovaSeq PE150 (NEB, Ipswich, MA, USA). After the successful construction of the library, initially, the library was quantified using a Qubit 4.0 fluorometer and size distribution by Agilent 2100 Bioanalyzer (Agilent Technologies, Santa Clara, CA, USA) and final quantification by qRT-PCR. The prepared libraries were sequenced by NovaSeq PE150 (Illumina, San Diego, CA, USA) by Novogene Technology Co., Ltd. (Beijing, China).

### 4.3. Data Processing and Genome Assembly

The raw sequencing data obtained were quality-checked using FastQC version 0.12.1. The low-quality reads were filtered out from the data using the Trimmomatic version 0.39 [63]. The de novo assembly of trimmed data (called clean data) was done using SPAdes version 4.0.0 [64]. The draft assemblies were processed further for gap filling using the GapCloser version 1.12, which filters the low-depth sequences (e.g., <0.35 average depth) and contigs with a size below 500 bp to get the final genome assemblies for further analysis.

### 4.4. Pangenome and Bioinformatics Analysis

The finally assembled genome was used to predict the coding genes by GeneMarkS (version 4.17) software (Available online: http://topaz.gatech.edu/GeneMark/ (accessed on 20 September 2024)) [65]. Gene functional annotation was completed by the Cluster of Orthologous Genes (COG) database [66]. Type N secretion systems (TNSS) were retrieved based on the protein functional database, and for Gram-negative bacteria such as *E. coli*, T3SS effector proteins were predicted by Effective T3 software (version 1.0.1) [67]. The comprehensive antibiotic resistance (CARD) database was used to identify the antibiotic resistance efflux pumps acquired by each strain [68]. The virulence determinants, prediction as a human pathogen, serotyping, and CHTyping were completed by VirulenceFinder 2.0 (specie *E. coli*, 95% ID threshold, 80% minimum length), PathogenFinder 1.1 (phylum = gamma proteobacteria), SerotypeFinder 2.0 (*E. coli*, 95% ID threshold, and 80% minimum length), and CHTyper 1.0 (95% ID threshold), respectively, under Center for Genomic Epidemiology (CGE) online server (Available online: https://genomicepidemiology.org/services/ (accessed on 15 August 2024)). The relationship of seven *E. coli* strains with verified human pathogenic strains from the NCBI database was completed by phylogeny construction using CSIPhylogeny 1.4 and visualized by TVBOT under the ChiPlot online server (Available online: https://www.chiplot.online/ (accessed on 10 October 2024)).

## 5. Conclusions

The present study investigated seven *E. coli* strains isolated from a dairy farm environment in Xinjiang Province, China, carrying wide antibiotic efflux-associated genes and virulence genes. Moreover, the study also noted that a few of the virulence genes were found to be associated with prophages and genomic islands, which may enhance their potential to be transferred to other bacterial species. Although this study was conducted on a single form, *it* might suggest that the dairy farm environment can be a potential reservoir of such genes in *E. coli*. This study served as a preliminary genomic characterization of MDR *E. coli* strains within a specific farming system to identify potential ARG reservoirs and strain diversity in a dairy environment. Even if the study has been performed on a limited number of strains, it highlights the genetic diversity and adaptability of *E. coli* strains, emphasizing their potential for antibiotic resistance and virulence. The study underscores the necessity for ongoing large-scale genomic surveillance and research to mitigate public health risks associated with these pathogens. The actual findings contribute valuable insights into the understanding of *E. coli* in various environments, reinforcing the importance of genomic characterization in addressing public health challenges.

## Figures and Tables

**Table 1:** Cluster of Orthologous Genes (COG) functional classification of seven *E. coli* strains.

Function Class	Number of Matched Genes, *n*						
	17XJ28	17XJ30	17XJ31	18XJ24	18XJ28	18XJ85	19XJ31
A	2	2	2	2	2	3	2
B	1	1	0	0	1	0	0
C	301	298	217	324	304	285	310
D	43	48	44	49	49	37	53
E	375	372	300	389	374	355	379
F	110	105	84	108	107	103	102
G	415	417	331	444	430	458	452
H	201	195	164	207	197	208	205
I	125	125	105	134	132	128	124
J	250	249	226	261	249	237	249
K	341	349	322	349	350	384	367
L	177	192	187	193	179	172	191
M	286	285	249	292	295	291	291
N	154	134	136	122	139	175	150
O	176	179	161	183	174	164	180
P	260	252	200	264	272	290	291
Q	84	85	77	85	90	102	84
R	325	312	275	336	320	343	336
S	228	228	209	244	221	272	233
T	232	229	191	234	230	233	236
U	79	98	94	69	92	94	97
V	113	112	111	116	116	138	130
W	44	46	48	39	51	48	42
X	138	142	218	128	111	215	138
Total	4460	4455	3951	4572	4485	4736	4647

A: RNA processing and modification; B: Chromatin structure and dynamics; C: Energy production and conversion; D: Cell cycle control, cell division, and chromosomal partitioning; E: Amino acid transport and metabolism; F: Nucleic acid transport and metabolism; G: Carbohydrate transport and metabolism; H: Coenzyme transport and metabolism; I: Lipid transport and metabolism; J: Translation, ribosomal structure, and biogenesis; K: Transcription; L: Replication, recombination, and repair; M: Cell wall/membrane/envelope biogenesis; N: Cell motility; O: Posttranslational modification, protein turnover, and chaperones; P: Inorganic transport and metabolism; Q: Secondary metabolites biosynthesis, transport, and catabolism; R: General function prediction only; S: Function unknown; T: Signal transduction mechanisms; U: Intracellular trafficking, secretion, and vesicular transport; V: Defense mechanisms; W: Extracellular structures; X: Mobilome: prophages, transposons.

**Table 2:** Prophage and genomic island-associated virulence genes (VGs).

Strain ID	Prophage-Associated VGs	Genomic Island-Associated VGs
17XJ28	*stx1*, *colE5*, *hha*, *iha*, *iss*, *ompT*	None
17XJ30	*hra*, *iss*, *papC*	None
17XJ31	*f17G*, *f17C*, *nlpI*	None
18XJ24	*iss*	*fimH*, *ompT*
18XJ28	*mcmA*	None
18XJ85	*iss*	None
19XJ31	*iss, nlpI*, *terC*	*afaA*, *afaB*, *afaC*, *afaD*, *iha*, *kpsE*

**Table 3:** Biofilm-associated genes acquired by seven MDR *E. coli* strains.

Strain ID	Biofilm Genes	Biofilm Genes Activated by Environmental Signals	sRNAs Involved in Biofilm Formation
17XJ28	*crr*, *cyaA*, *crp*, *flhD*, *flhC*, *flgM*, *fliA*, *fliZ*, *yhjH*, *yegE*, *ycgR*, *yciR*, *ydaM*, *mlrA*, *rpoS*, *adrA*, *adrB*, *bcsA*, *csgA*, *csgB*, *csgD*, *luxS*, *lsrR*, *wza*, *BarA*, *SdiA*, *uvrY*, *csrA*, *glgA*, *glgC*, *glgP*, *pgaA*, *pgaB*, *pgaC*, *pgaD*	*oxyR*, *arcB*, *arcA*, *flhD*, *flhC*, *rcsA*, *rcsB*, *rcsC*, *rcsD*, *gcvA*, *gcvR*, *envZ*, *ompR*, *crp*, *rpoS*, *ydaM*, *csgD*	None
17XJ30	*crr*, *cyaA*, *crp*, *flhD*, *flhC*, *flgM*, *fliA*, *fliZ*, *yhjH*, *yegE*, *ycgR*, *yciR*, *ydaM*, *mlrA*, *rpoS*, *adrA*, *adrB*, *bcsA*, *csgA*, *csgB*, *csgD*, *luxS*, *lsrR*, *wza*, *BarA*, *SdiA*, *uvrY*, *csrA*, *glgA*, *glgC*, *glgP*, *pgaA*, *pgaB*, *pgaC*, *pgaD, dksA, ag43*	*oxyR*, *arcB*, *arcA*, *flhD*, *flhC*, *rcsA*, *rcsB*, *rcsC*, *rcsD*, *gcvA*, *gcvR*, *envZ*, *ompR*, *crp*, *rpoS*, *ydaM*, *csgD*	None
17XJ31	*crr*, *cyaA*, *crp*, *flhD*, *flhC*, *flgM*, *fliA*, *fliZ*, *yhjH*, *yegE*, *ycgR*, *yciR*, *ydaM*, *mlrA*, *rpoS*, *adrA*, *adrB*, *bcsA*, *csgA*, *csgB*, *csgD*, *luxS*, *lsrR*, *wza*, *BarA*, *SdiA*, *uvrY*, *csrA*, *glgA*, *glgC*, *glgP*, *pgaA*, *pgaB*, *pgaC*, *pgaD, dksA, ag43*	*oxyR*, *arcB*, *arcA*, *flhD*, *flhC*, *rcsA*, *rcsB*, *rcsC*, *rcsD*, *gcvA*, *gcvR*, *envZ*, *ompR*, *crp*, *rpoS*, *ydaM*, *csgD*	None
18XJ24	*crr*, *cyaA*, *crp*, *flhD*, *flhC*, *flgM*, *fliA*, *fliZ*, *yhjH*, *yegE*, *ycgR*, *yciR*, *ydaM*, *mlrA*, *rpoS*, *adrA*, *adrB*, *bcsA*, *csgA*, *csgB*, *csgD*, *luxS*, *lsrR*, *wza*, *BarA*, *SdiA*, *uvrY*, *csrA*, *glgA*, *glgC*, *glgP*, *pgaA*, *pgaB*, *pgaC*, *pgaD*	*oxyR*, *arcB*, *arcA*, *flhD*, *flhC*, *rcsA*, *rcsB*, *rcsC*, *rcsD*, *gcvA*, *gcvR*, *envZ*, *ompR*, *crp*, *rpoS*, *ydaM*, *csgD*	None
18XJ28	*crr*, *cyaA*, *crp*, *flhD*, *flhC*, *flgM*, *fliA*, *fliZ*, *yhjH*, *yegE*, *ycgR*, *yciR*, *ydaM*, *mlrA*, *rpoS*, *adrA*, *adrB*, *bcsA*, *csgA*, *csgB*, *csgD*, *luxS*, *lsrR*, *wza*, *BarA*, *SdiA*, *uvrY*, *csrA*, *glgA*, *glgC*, *glgP*, *pgaA*, *pgaB*, *pgaC*, *pgaD, dksA, ag43*	*oxyR*, *arcB*, *arcA*, *flhD*, *flhC*, *rcsA*, *rcsB*, *rcsC*, *rcsD*, *gcvA*, *gcvR*, *envZ*, *ompR*, *crp*, *rpoS*, *ydaM*, *csgD*	None
18XJ85	*crr*, *cyaA*, *crp*, *flhD*, *flhC*, *flgM*, *fliA*, *fliZ*, *yhjH*, *yegE*, *ycgR*, *yciR*, *ydaM*, *mlrA*, *rpoS*, *adrA*, *adrB*, *bcsA*, *csgA*, *csgB*, *csgD*, *luxS*, *lsrR*, *wza*, *BarA*, *SdiA*, *uvrY*, *csrA*, *glgA*, *glgC*, *glgP*, *pgaA*, *pgaB*, *pgaC*, *pgaD, dksA, ag43*	*oxyR*, *arcB*, *arcA*, *flhD*, *flhC*, *rcsA*, *rcsB*, *rcsC*, *rcsD*, *gcvA*, *gcvR*, *envZ*, *ompR*, *crp*, *rpoS*, *ydaM*, *csgD*	None
19XJ31	*crr*, *cyaA*, *crp*, *flhD*, *flhC*, *flgM*, *fliA*, *fliZ*, *yhjH*, *yegE*, *ycgR*, *yciR*, *ydaM*, *mlrA*, *rpoS*, *adrA*, *adrB*, *bcsA*, *csgA*, *csgB*, *csgD*, *luxS*, *lsrR*, *wza*, *BarA*, *SdiA*, *uvrY*, *csrA*, *glgA*, *glgC*, *glgP*, *pgaA*, *pgaB*, *pgaC*, *pgaD*	*oxyR*, *arcB*, *arcA*, *flhD*, *flhC*, *rcsA*, *rcsB*, *rcsC*, *rcsD*, *gcvA*, *gcvR*, *envZ*, *ompR*, *crp*, *rpoS*, *ydaM*, *csgD*	None

## Data Availability

The raw data supporting the conclusions of this article will be made available by the authors upon request. The whole genome sequences of the seven *E. coli* strains analyzed in this study are available from the NCBI under BioProject ID: PRJNA1049405. The accession numbers of seven *E. coli* genome data are JAYKSK000000000, JAYJLB000000000, JBDYKH000000000, JAYKSJ000000000, JAYJLA000000000, JBDYKG000000000, and JAYKSI000000000 (accessed on 21 January 2025).

## Abbreviations

The following abbreviations are used in this manuscript:

TNSS	Type N secretion systems
CGE	Center for Genomic Epidemiology
WGS	Whole Genome Sequencing
COG	Cluster of Orthologous Genes
EMB	Eosin Methylene Blue
VGs	Virulence genes
MDR	Multidrug resistance
STEC	Shiga toxin-producing *E. coli*
CCCP	Carbonyl-cyanide m-chlorophenylhydrazone
VTEC	Verotoxigenic *E. coli*
APEC	Avian pathogenic *E. coli*
ETEC	Enterotoxigenic *E. coli*
EHEC	Enterohemorrhagic *E. coli*
NMEC	Neonatal meningitis *E. coli*
UPEC	Uropathogenic *E. coli*
EAEC	Enteroaggregative *E. coli*
Ex-PEC	Extraintestinal pathogenic *E. coli*
DEC	Diarrheagenic *E. coli*
MGE	Mobile genetic elements
*E. coli*	*Escherichia coli*
GIs	Genomic islands
ARGs	Antibiotic resistance genes
